# A Continuous Correlated Beta Process Model for Genetic Ancestry in Admixed Populations

**DOI:** 10.1371/journal.pone.0151047

**Published:** 2016-03-11

**Authors:** Zachariah Gompert

**Affiliations:** Department of Biology, Utah State University, Logan, UT, United States of America; Universitat Pompeu Fabra, SPAIN

## Abstract

Admixture and recombination create populations and genomes with genetic ancestry from multiple source populations. Analyses of genetic ancestry in admixed populations are relevant for trait and disease mapping, studies of speciation, and conservation efforts. Consequently, many methods have been developed to infer genome-average ancestry and to deconvolute ancestry into continuous local ancestry blocks or tracts within individuals. Current methods for local ancestry inference perform well when admixture occurred recently or hybridization is ongoing, or when admixture occurred in the distant past such that local ancestry blocks have fixed in the admixed population. However, methods to infer local ancestry frequencies in isolated admixed populations still segregating for ancestry do not exist. In the current paper, I develop and test a continuous correlated beta process model to fill this analytical gap. The method explicitly models autocorrelations in ancestry frequencies at the population-level and uses discriminant analysis of SNP windows to take advantage of ancestry blocks within individuals. Analyses of simulated data sets show that the method is generally accurate such that ancestry frequency estimates exhibited low root-mean-square error and were highly correlated with the true values, particularly when large (±10 or ±20) SNP windows were used. Along these lines, the proposed method outperformed *post hoc* inference of ancestry frequencies from a traditional hidden Markov model (i.e., the linkage model in structure), particularly when admixture occurred more distantly in the past with little on-going gene flow or was followed by natural selection. The reliability and utility of the method was further assessed by analyzing genetic ancestry in an admixed human population (Uyghur) and three populations from a hybrid zone between *Mus domesticus* and *M. musculus*. Considerable variation in ancestry frequencies was detected within and among chromosomes in the Uyghur, with a large region of excess French ancestry harboring a gene with a known disease association. Similar variation was detected in the mouse hybrid zone, with notable constancy in regions of excess ancestry among admixed populations. By filling what has been an analytical gap, the proposed method should be a useful tool for many biologists. A computer program (popanc), written in C++, has been developed based on the proposed method and is available on-line at http://sourceforge.net/projects/popanc/.

## Introduction

Genetic admixture between differentiated populations or species is common in plants and animals [1–6], including humans [7–10]. Admixture and recombination result in individuals whose genomes comprise a mosaic of chromosome segments with different genetic ancestry, that is to say, chromosome segments that have been inherited from different source populations. Given the prevalence of admixture, analyses of genetic ancestry are relevant in many areas of biology [11, 12]. For example, patterns of admixture and introgression in the wild show that species boundaries are often porous, and have been used to characterize the genetic basis of adaptation and reproductive isolation [13–19]. Patterns of genetic variation in admixed populations also provide information about past demographic events [11, 20–22]. Moreover, an accurate characterization of genetic ancestry is required when using genome-wide association or admixture mapping to identify genetic variants associated with trait variation or disease [11, 23–27]. Finally, admixture can be catalyzed by anthropogenic habitat alteration or species introductions, and can cause species collapse or extinction [28–31]. Thus, an understanding of admixture can be important for biodiversity conservation and wildlife management.

Numerous statistical methods have been developed to infer genetic ancestry from molecular data (reviewed in [12]). Early methods considered unlinked genetic markers and were primarily concerned with inference of genome-average ancestry, that is, the proportion of an individual’s genome inherited from each of *K* potential source populations (as in the admixture model in structure [23]). More recently, a variety of methods have been proposed to resolve genetic ancestry into a series of continuous blocks of DNA inherited from different source populations, and thereby infer local or locus-specific ancestry along chromosomes [32–37]. Local ancestry inference can be based on population allele frequencies or haplotypes. Hidden Markov models (HMMs) are commonly used to model correlations in local ancestry along chromosome (as in the linkage HMM in structure), and in some cases, background linkage disequilibrium (this includes Markov-HMMs and infinite-HMMs as in sabre and mspectrum) [32, 33, 36]. Local ancestry inference can be very accurate, particularly when samples from well-defined source populations and phased DNA sequence data are available [35–37]. Methods that summarize genetic ancestry for a population or lineage also exist. These include tree-based methods used to infer population admixture proportions [7, 38] and genomic cline models, which can be used to quantify differential introgression in hybrid zones [39, 40].

Different ancestry inference methods are better suited for different tasks or under different conditions. For example, estimates of genome-average ancestry from structure can be used to identify recent hybrids [41], whereas tree-based methods are better able to detect ancient introgression [7, 38]. My primary focus in this paper is on local ancestry inference at the population-level. Because of recombination, genetic drift and selection in admixed populations, population local ancestry frequencies can vary across the genome [15, 39, 42–44]. In other words, local ancestry from a given source population can be more common in some regions of the genome than others. Such variation in local ancestry frequencies precedes genome stabilization during hybrid speciation [11], and has been associated with adaptation in several systems, including maize [19], humans [45], and butterflies [17, 18]. Several approaches have been used to quantify variation in local ancestry frequencies. In particular, ancestry frequencies can be inferred *post hoc* from resolved local ancestry blocks [19], or in the case of very ancient admixture, using tree-based methods [17]. Similarly, genomic cline methods can provide derived summaries of local ancestry frequencies when hybridization is an ongoing process [39, 40].

Herein, I propose and evaluate a new statistical method to estimate local ancestry frequencies. The primary motivation for this method is a desire to infer ancestry frequencies in admixed populations when ongoing gene flow from source populations is rare or absent, but before genome stabilization is complete. Such situations exists in nature [6, 46], but do not meet the assumptions of existing methods (local ancestry inference generally assumes ancestry frequencies do not vary across the genome and tree-based methods ignore segregating variation within lineages). Moreover, analyses of ancestry frequencies in isolated admixed populations should provide novel insights about the relative roles of selection, drift and recombination in shaping genomes (e.g., [47]), and on the genetic basis of trait variation (e.g., [27]). I first describe the proposed method, which combines discriminant analysis with a continuous correlated beta process model to jointly estimate local ancestry within individuals and local ancestry frequencies at the population-level. I then assess the accuracy of the method by applying it and a traditional HMM approach to simulated data sets. The reliability and utility of the method is further demonstrated by using it to analyze genetic ancestry in an admixed human population (Uyghur), and three admixed populations from a house mouse hybrid zone. These analyses show that the method is both accurate and useful. Computer software implementing these methods (popanc) is available on-line at http://sourceforge.net/projects/popanc/.

## Methods

### Model

**Fig 1:**
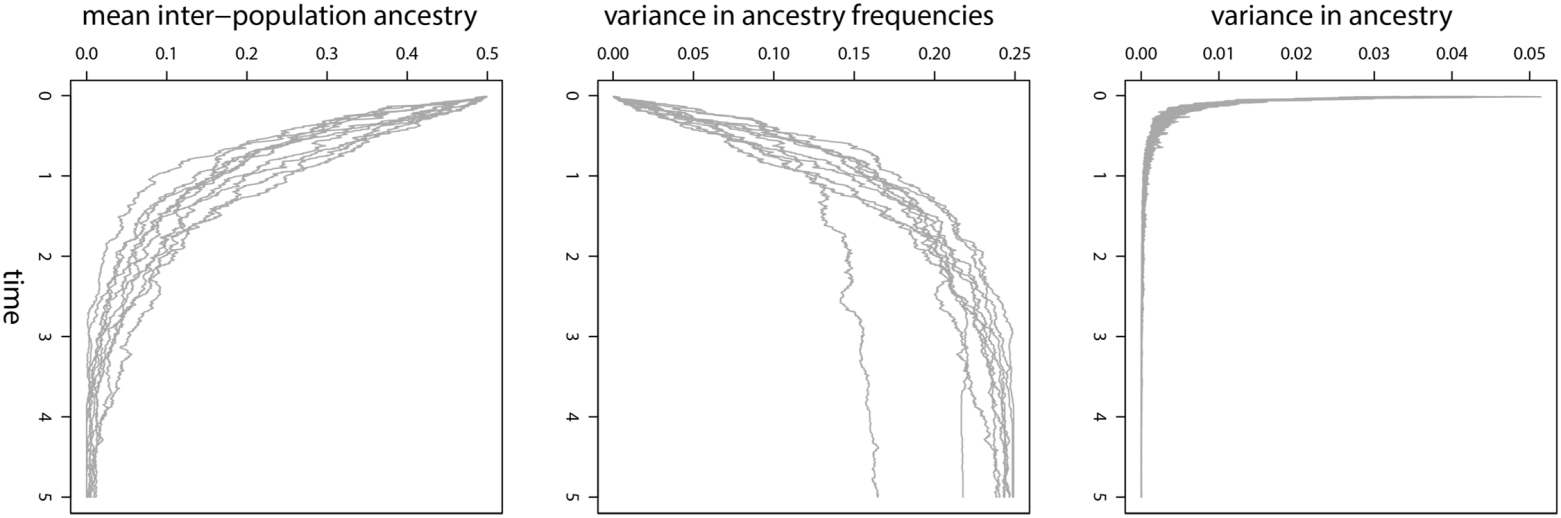
Variance in ancestry. Plots depict summaries of genetic ancestry from 10 replicate simulations (gray lines). Each simulation followed a Wright-Fisher model starting with an admixed population composed entirely of F1s. The population then evolved by recombination and genetic drift. Population size was constant (2*N* = 200), and simulations ran for *t* = 1000 generation (time is reported relative to populations size as *t*/2*N*). Ancestry was followed at 100 genetic loci that were equally spaced on a 1 Morgan chromosome. Plots show the variance in genome-average ancestry among individuals (top pane), the variance in local ancestry frequencies among loci, and the proportion of loci where individuals are expected to have one gene copy from each source population (i.e., inter-population ancestry [6]). Note that variation in ancestry frequencies persists long-after variation in genome-average ancestry has decayed.

As a basis for the proposed statistical method, consider a model where admixture between two populations, *A* and *B*, occurs *t* generations in the past. The resulting admixed population then evolves until the present by recombination, drift, and selection, but with little or no ongoing gene flow from the source populations (as in [48, 49]). Under this model, genome-average ancestry should initially vary among individuals because of variation in the number of migrant ancestors (genealogy variance) and variation in the contribution of genetic material from each ancestor (assortment variance) (Fig 1, [21]). However, these sources of variation should decay rapidly, and be replaced by genome-wide variation in local ancestry frequencies among chromosome segments [44]. Eventually, chromosome segments will fix for local ancestry from population *A* or *B*. This process has been referred to as genome stabilization, particularly in the context of homoploid hybrid speciation [11, 50]. My current focus is on inference during the intermediate stages of this processes, that is, once variation in genome-average ancestry has mostly been removed, but while variation for local ancestry is still segregating in the admixed population (i.e., before genome stabilization; Fig 1). Once genome stabilization is complete or nearly complete, tree-based methods can be used to analyze local ancestry at a population or species-level, as intra-population variation in genetic ancestry can be ignored. However, during this intermediate period, methods are needed that allow for variable ancestry frequencies and that can account for genetic divergence in the admixed population (via drift or selection).

Most traditional methods for local ancestry inference use homogeneous HMMs or extensions of these [32, 33, 36]. HMMs are parameterized by a transition probability matrix that gives the probability of switching from one ancestry state (or ancestral haplotype) to another as one moves along a chromosome. Homogeneous HMMs assume that the transition probability matrix is constant and independent of the position in the genome. In essence, this assumes that local ancestry frequencies are the same everywhere in the genome. Although this assumption is reasonable when admixture occurred recently or when hybridization is ongoing, it becomes less tenable as progress towards genome stabilization occurs (Fig 1). This problem could be circumvented by defining a non-homogeneous HMM where the transition probability matrix varies along chromosomes to reflect variation in local ancestry frequencies, but computational methods for non-homogeneous HMMs are not well developed. Instead, my proposed method uses a continuous correlated beta process model (CCBPM; [51]) to co-estimate local ancestry within individuals and population-level local ancestry frequencies while explicitly modeling the genomically autocorrelated variation in the latter. Key model parameters can be estimated in a computationally efficient way via Gibbs sampling. An additional advantage of this method relative to many HMMs is that inference does not depend on phased data. While such data might be available for humans and some model systems, sequencing strategies commonly used in non-model organisms, such as genotyping-by-sequencing (GBS) methods [18, 52, 53], generate sparse, un-phased SNP data. My goal is to develop a method that can be used in these situations, as long as a draft reference genome is available.

First, I begin with a general description of a CCBPM. Consider a series of binomial experiments where the probability of success (*θ*) varies from experiment to experiment. In a Bayesian framework, it would be natural to place a conjugate beta prior on the probability of success for each experiment (*θ*_*x*_), and thereby obtain the posterior probability distribution for *θ*_*x*_ (also a beta distribution), which would be $\text{Pr}\left(\theta_{x}|y_{x},n_{x},\alpha_{0},\beta_{0}\right)\propto \theta_{x}^{y_{x}+\alpha_{0}-1}{\left(1-\theta_{x}\right)}^{n_{x}-y_{x}+\beta_{0}-1}$, where *y*_*x*_ denotes the number of successes out of *n*_*x*_ trials, and *α*_0_ and *β*_0_ are shape parameters for the beta prior. Note that the posterior is a beta distribution with shape parameters *α* = *y*_*x*_ + *α*_0_ and *β* = *n*_*x*_ − *y*_*x*_ + *β*_0_. Now assume that experiments are conducted one after another and that the probability of success is autocorrelated in time (or space) such that successive experiments have similar values of *θ* (i.e., *θ*_*x*_ and *θ*_*x*+1_ tend to be similar). While the model described above could still be used for inference, it would be sub-optimal as it does not provide a means to share information about *θ* among experiments. An alternative solution is to estimate *θ* using a CCBPM [51], which is a graphical model that generalizes the Bayesian model above by allowing for information sharing among experiments. A kernel function *K*(*x*, *x*′) dictates the extent that information is shared among experiments. Different kernel functions are possible, but the kernel should be a decreasing function of the time or distance between a pair of experiments. Under this model an approximate posterior distribution for each *θ*_*x*_ can be generated by drawing samples from Pr(*θ*_*x*_|**y**, **n**, *α*_0_, *β*_0_) = beta(*α* = (∑_*i*_ *y*_*i*_ *k*(*x*, *i*)) + *α*_0_, *β* = (∑_*i*_(*n*_*i*_ − *y*_*i*_)*k*(*x*, *i*)) + *β*_0_).

As the general description above makes clear, a CCBPM can naturally be used to model local ancestry frequencies in an admixed population. In particular, at each locus (*x*) the number of gene copies with ancestry from source population *A* (*z*_*x*_) in a sample of *n*_*x*_ diploid individuals can be modeled as the outcome of a binomial experiment with the probability of success given by the population ancestry frequencies at that locus (*q*_*x*_ and 1 − *q*_*x*_). Because of recombination and the chromosomal nature of inheritance, ancestry frequencies should be autocorrelated along chromosomes. The CCBPM is used to share information across linked loci when estimating each *q*_*x*_. Thus, the approximate conditional posterior distribution for each *q*_*x*_ is,

$$\begin{array}{ccc} \text{Pr}(q_{x}|z,2n,\alpha_{0},\beta_{0}) & = & \text{beta}(\alpha =(\sum_{i}z_{i}k\left(x,i\right))+\alpha_{0},\beta =(\sum_{i}\left(2n_{i}-z_{i}\right)k\left(x,i\right))+\beta_{0})\\ & \propto & q_{x}^{\alpha_{0}-1+\sum_{i}z_{i}k\left(x,i\right)}{\left(1-q_{x}\right)}^{\beta_{0}-1+\sum_{i}\left(2n_{i}-z_{i}\right)k\left(x,i\right)}. \end{array}$$ (1)

Here I use the squared exponential kernel $k\left(x,i\right)=\text{exp}(\frac{-{\left(x-i\right)}^{2}}{\sigma })$, where *σ* is a scale parameter. While the scale parameter could be fixed, I instead propose to place an uniform prior on this parameter and estimate it from the data (though my main aim is to integrate over uncertainty in this nuisance parameter rather than make inferences about it).

In this model, local ancestry (**z**) is not an observed quantity, but instead represents a latent variable that must be inferred from the data. *z*_*x*_ can be decomposed as *z*_*x*_ = ∑_*j*_ *z*_*xj*_ where the sum is over individuals and *z*_*xj*_ ∈ {0, 1, 2} denotes the number of gene copies at locus *x* that individual *j* inherited from source population *A*. Following Bayes’ rule, a posterior distribution for *z*_*xj*_ can be specified as,

$$\begin{array}{c} \text{Pr}\left(z_{xj}|s_{xj},q_{x}\right)\propto \text{Pr}\left(s_{xj}|z_{xj}\right)\text{Pr}\left(z_{xj}|q_{x}\right) \end{array}$$ (2)

where *s*_*xj*_ is the DNA sequence data (discussed more below). The first term on the right side of Eq (2) represents the probability of the observed sequence data for individual *j* conditional on that individual having 0, 1 or 2 gene copies derived from source population *A*, and the last term is the prior probability of inheriting *z*_*xj*_ gene copies from source population *A*. The latter is clearly given by,

$$\begin{array}{c} \text{Pr}\left(z_{xj}|q_{x}\right)∼\text{binomial}(q_{x},2), \end{array}$$ (3)

but specifying a probability distribution for the first term (i.e., the likelihood of *z*_*xj*_ given the data) is more complicated. Thus, I will describe my approach for specifying Pr(*s*_*xj*_|*z*_*xj*_) in detail. Because of segregating allelic variation in the source populations and drift (or selection) in the admixed and source populations, sequence data from any particular nucleotide variant (i.e., SNP) can be rather uninformative about local ancestry. To overcome this limitation and to model expected autocorrelations in local ancestry within individuals, I propose to approximate Pr(*s*_*xj*_|*z*_*xj*_) using discriminant analysis (DA; a related approach was used by [54] for global ancestry inference).

Here DA is used to provide different weights to different SNPs such that they are maximally informative about local ancestry. The analysis proceeds one SNP or locus at a time. First, a window is defined around each genetic locus; a window includes a specific number of neighboring SNPs (this could be constrained by physical or recombination distance). Choosing a reasonable window size can be important (this is discussed more in the Discussion). In particular, larger windows will contain more information about local ancestry, but if windows become too large they will frequently span ancestry breakpoints, which is undesirable (see e.g., [34, 55]). Also, the window cannot include more SNPs than reference individuals (i.e., the number of observations must exceed the number of variables). DA is then used to generate a discriminant function and thereby distinguish between individuals with 0, 1 or 2 gene copies from source population *A* for each window (i.e., to infer *z*_*xj*_). A set of reference samples from each source population (i.e., populations *A* and *B*) is required to generate the discriminant function. These represent *z*_*x*_ = 0 (source *B*) and *z*_*x*_ = 2 (source *A*). Reference samples with one gene copy from each source population can then be simulated to represent *z*_*x*_ = 1. My implementation of DA then proceeds as follows. Let *S*_*x*_ be a *N* × *P* matrix with reference individuals as rows and the genotypic data from the set of genetic variants within a window as columns. Here the genotypic data are centered counts of one of the two alleles for each SNP. The within group covariance matrix (*S*_*w*_) is then calculated as,

$$\begin{array}{c} S_{w}=\frac{\sum_{g}\left(n_{g}-1\right)s_{xg}^{T}s_{xg}}{\sum_{g}n_{g}-3} \end{array}$$ (4)

where the summation is over the three groups (i.e., samples with 0, 1 or 2 gene copies from source population *A*), *n*_*g*_ is the sample size for group *g*, and *s*_*xg*_ is the sub-matrix containing only individuals from group *g*. Next, the between group scatter matrix (*S*_*B*_) is obtained as,

$$\begin{array}{c} S_{v}=\frac{1}{3}\sum_{g}\left(\mu_{g}-\mu \right){\left(\mu_{g}-\mu \right)}^{T} \end{array}$$ (5)

where *μ*_*g*_ and *μ* are the group and grand means of *s*_*xg*_ and *S*_*x*_, respectively. Eigenvalue decomposition of the canonical matrix $S_{w}^{-1}S_{b}$ can then be used to obtain the discriminant function. Specifically, the eigenvector associated with the largest eigenvalue of the canonical matrix contains the discriminant coefficients. These coefficients can be used to calculate a discriminant score for each reference sample and thereby transform the reference samples onto a new space that maximizes the genetic differences among the three groups relative to within group variation. Note that only the first discriminant function is required to separate the three groups because the *z*_*x*_ = 1 reference group is intermediate between the two other groups.

The mean and variance of the discriminant scores for the reference individuals from each group are used to define Pr(*s*_*xj*_|*z*_*xj*_) such that,

$$\begin{array}{c} \text{Pr}\left(s_{xj}|z_{xj}\right)=\text{Pr}\left(d_{xj}=f\left(s_{xj}\right)|z_{xj}\right)=\text{normal}(\mu ={\bar{d}}_{xg=z_{x}},\sigma^{2}=\text{var}\left(d_{xg=z_{x}}\right)), \end{array}$$ (6)

where ${\bar{d}}_{xg=z_{x}}$ and *var*(*d*_*xg* = *z*_*z*__) are the mean and variance of the discriminant scores for the set of reference samples with ancestry *z*_*x*_, *f*(*s*_*xj*_) is the discriminant function, and *d*_*xj*_ is the discriminant score for an individual with unknown ancestry. Thus, after transforming the admixed individuals onto the new sample space with the discriminant function developed from the reference set, Eq (6) can be used to calculate the probability of the sequence data (or more precisely the probability of the discriminant score based on the sequence data) if the individual has 0, 1 or 2 gene copies from source population *A*.

A computer program (popanc), written in C++, has been developed to generate parameter estimates from the model described above. The program first performs the DA using linear algebra functions provided by the GNU Scientific Library [56]. Markov chain Monte Carlo (MCMC) is then used to obtain samples from the approximate posterior distributions for each of the model parameters (this is an approximation because Eq (1) represents a process-model generalization of Bayes’ rule [51]). MCMC includes Gibbs samplers for population ancestry frequencies (**q**) based on Eq (1) and individual local ancestry (**z**) based on Eqs (3) and (6). A Metropolis update is performed for the scale parameter *σ*. HDF5 is used for efficient storage and processing of the MCMC samples [57].

### Simulations and data analysis

Multiple data sets were simulated and analyzed to assess the efficacy of the proposed method. Simulations were individual-based, and tracked ancestry segments rather than genetic markers (as in [11, 39, 40]). In each simulation an admixed population of *N* diploid individuals was initiated via hybridization between two source populations, *A* and *B*. This was followed by *t* discrete generations, where mating occurred within the admixed population with or without ongoing gene flow (*m*). Genomes consisted of 2, 1 Morgan chromosomes (i.e., 1 recombination event occurred per chromosome each generation). All simulations included genetic drift and some also included natural selection (details below). At the end of each simulation, 50 individuals were sampled from each source population and the admixed population, and genetic marker (SNP) data were generated for these individuals. Allele frequencies in the source populations were drawn from independent uniform distributions bounded by 0.05 and 0.95. Genotypes were then obtained by randomly sampling from the population allele frequencies. A similar procedure was used to generate genotypes for the admixed individuals, except alleles were sampled based on the source population allele frequencies and local ancestry. Moreover, allele frequencies within ancestry segments were modified to account for genetic drift (genetic drift affects ancestry frequencies, and also allele frequencies within ancestry segments). Specifically, for each genetic marker and ancestry type (source *A* and *B*) a new allele frequency was sampled from beta(*α* = *pγ*, (1 − *p*)*γ*), where $\gamma =-1\frac{F-1}{F}$ and $F=-\text{exp}(-\frac{t}{N})(\text{exp}(\frac{t}{N})-1)$ [58, 59]. Evolutionary dynamics depend on the ratio of the time since admixture and the population size (i.e., $\frac{t}{N}$). Allowing drift to affect ancestry and allele frequencies (rather than just ancestry frequencies) is realistic and important as it makes local ancestry inference considerably more difficult.

A series of data sets was simulated to determine how time since admixture affects the accuracy of ancestry frequency estimates. Ten replicate data sets were simulated with an admixed population size of *N* = 500 and *t* = 20, 50, or 200 generations since admixture ($\frac{t}{N}=0.04$, 0.10, or 0.40) with *m* = 0 (no ongoing gene flow). Another series of simulations was used to quantify the effect of selection on ancestry inference. Here, each individual’s fitness was determined by its ancestry at *L* loci, with individuals having mixed ancestry at these loci suffering reduced fitness (i.e., underdominance was assumed). Fitness was multiplicative such that $w_{j}={\left(1-s\right)}^{l_{j}}$, where *w*_*j*_ is the relative fitness of individual *j* and *s* is the selection coefficient, and *l*_*j*_ is the number of loci (out of *L*) where individual *j* has one allele copy from each source population. Ten replicate data sets were generated with diffuse (*s* = 0.03 and *L* = 20 with 10 underdominant loci per chromosome) or strong (*s* = 0.3 and *L* = 2 with both underdominant loci on the same chromosome) selection. A third series of data sets was simulated with low (*m* = 0.005) or high (*m* = 0.05) rates of ongoing gene flow following the initial admixture event. Here *m* denotes the proportion of the admixed population composed of immigrants each generation. Ten replicate data sets were simulated for each migration rate and *t* = 200 generations with no selection.

The proposed method was then used to estimate population ancestry frequencies for each simulated data set. Data sets were analyzed using local ancestry windows that included 4, 10, or 20 SNPs on either side of the focal SNP. Two MCMC runs were conducted for each data set and window size, each with a 10,000 iteration burn-in, 30,000 total MCMC steps and a thinning interval of 5. Likely convergence to the stationary and adequate MCMC mixing were evaluated by calculating the Gelman and Rubin’s potential scale reduction factor and the effective sample size for each parameter. As a comparison, each data set was also analyzed using the correlated local ancestry HMM (i.e., linkage model) implemented in structure (version 2.3.4 [32]). Unlike many local ancestry inference approaches, the linkage model in structure can analyze un-phased genotypic data, and thus represents a valid comparison to the proposed method. Posterior estimates of local ancestry were based on two MCMC runs each with 10,000 iterations for sampling and 10,000 iteration burn-ins. Reference samples were specified, and were used as the sole source of information to infer source population allele frequencies. Population ancestry frequencies were then inferred *post hoc* from local ancestry estimates by equating the sample mean with the population ancestry frequency.

Approximate posterior distributions for population ancestry frequencies from the proposed CCBPM were summarized by calculating the posterior mean (point estimate) and 95% equal-tail probability intervals (ETPIs). Discrepancies between true ancestry frequencies and estimates from both the proposed method (posterior mean) and the linkage model in structure were quantified by calculating the root-mean-square deviation (RMSD). The coefficient of variation (CV) of the RMSD was then obtained by dividing the RMSD by the true parameter value. In addition, the CVRMSD was calculated for a null model where genome-average ancestry (calculated as the average of the local ancestry frequencies) was used as the estimate of the ancestry frequency for each locus. Model adequacy was also assessed by determining the frequency with which the true population ancestry frequency was included in the 95% ETPIs and by calculating the correlation between the true and estimated parameter values.

### Application to human genetic data

The proposed method was also applied to the Uyghur, which are a human population in Xinjiang, China known to be historically admixed with western Eurasian and Asian ancestry [7, 60]. Published results suggest that admixture in this group occurred approximately 790 (±60) years ago, or about 27 generations ago (assuming 29 years per generation, [7]; an alternative approach suggests admixture occurred 126 generations ago [60]). This places the time since admixture within the realm where the proposed methods should be applicable. Previous results indicate that the population admixture proportion for Uyghur (i.e., genome-average ancestry at the population-level) is 45.2% to 52.5% west Eurasian [7, 60, 61].

The data analyzed here come from the curated version of the Harvard HGDP-CEPH Genotypes for Population Genetics Analyses Supplement 10 that was released with admixtools [7]. Han (32 individuals) and French (26 individuals) were chosen as source populations for Uyghur (as suggested by [7]). The full data set of 621,038 SNPs was filtered to retain only those SNPs that were variable in the source populations with a minor allele frequency greater than 0.1, and to discard tightly linked, redundant SNPs (every third SNP was retained). This left a data set of 116,871 SNPs across the 22 human autosomes, which I analyzed with the proposed CCBPM. Each chromosome was analyzed separately for computational efficiency. Parameter estimates were obtained from two MCMC runs, each consisting of 30,000 iterations, a 10,000 iteration burn-in and a thinning interval of 5. I used a 15 SNP window, which previous results suggest should rarely span ancestry breakpoints (average ancestry block lengths are 2.43 to 4.07 cM [61]).

### Application to a mouse hybrid zone

The house mouse species *Mus domesticus* and *M. musculus* diverged about 500,000 years ago [62, 63], but now hybridize along a narrow hybrid zone in central Europe that formed a few thousand years ago [64]. Weak assortative mating and reduced hybrid fertility, particularly in males, limit gene flow across the hybrid zone, although the severity of each varies among populations and individuals (e.g., [65–67]). Because this hybrid zone is wide relative to dispersal distance, the hybrid zone consists of numerous admixed populations that differ in their genome composition but exhibit relatively little variation in genome-average ancestry within populations [67, 68]. Given these low dispersal rates (i.e., limited ongoing gene flow), ancestry frequencies as inferred from the proposed CCBPM should be a useful summary of genetic ancestry in these admixed populations. I focused on three admixed populations from the Bavarian transect through this hybrid zone, which differ in the genomic contribution of *M. domesticus* (as measured by a hybrid index, *h*): Tütenhausen (TU: $\bar{h}=0.27$, range = [0.18 − 0.36]), Haindlfing (HA: $\bar{h}=0.25$, range = [0.17 − 0.38]), and Neufahrn bei Freising (FS: $\bar{h}=0.58$, range = [0.45 − 0.68]) [16, 67, 69].

The data analyzed here are from captive-bred first generation offspring of wild-caught mice (only mice with both parents from a single locality were included; [69]); this included 21 mice from TU, 32 from HA, and 31 from FS. Smaller sample sizes were available for source (i.e., reference) populations: five *M. domesticus* from SO and ST (Pelka and Pallhausen) and five *M. musculus* from GL, RE and RF (Emling, Neufahrn bei Erding, and Finsingermoos) [67, 69]. The genetic data comprised 93,699 SNPs (from the Mouse Diversity Genotyping Array) from the 23 autosomal chromosomes (SNPs with very high LD were not included in the data set [69]). I analyzed each admixed populations separately using the proposed CCBPM. Parameter estimates were obtained from two MCMC runs, each consisting of 30,000 iterations, a 10,000 iteration burn-in and a thinning interval of 5. A window size of 4 SNPs was used because of the age of the hybrid zone and to account for the low parental sample sizes (i.e., to ensure that the sample size exceeded the number of variables for the DA). A key strength of this data set was that it allowed me to contrast patterns of variation in ancestry frequencies across multiple admixed populations, and thus ask about the consistency of these patterns.

## Results

### Performance on simulated data

**Fig 2:**
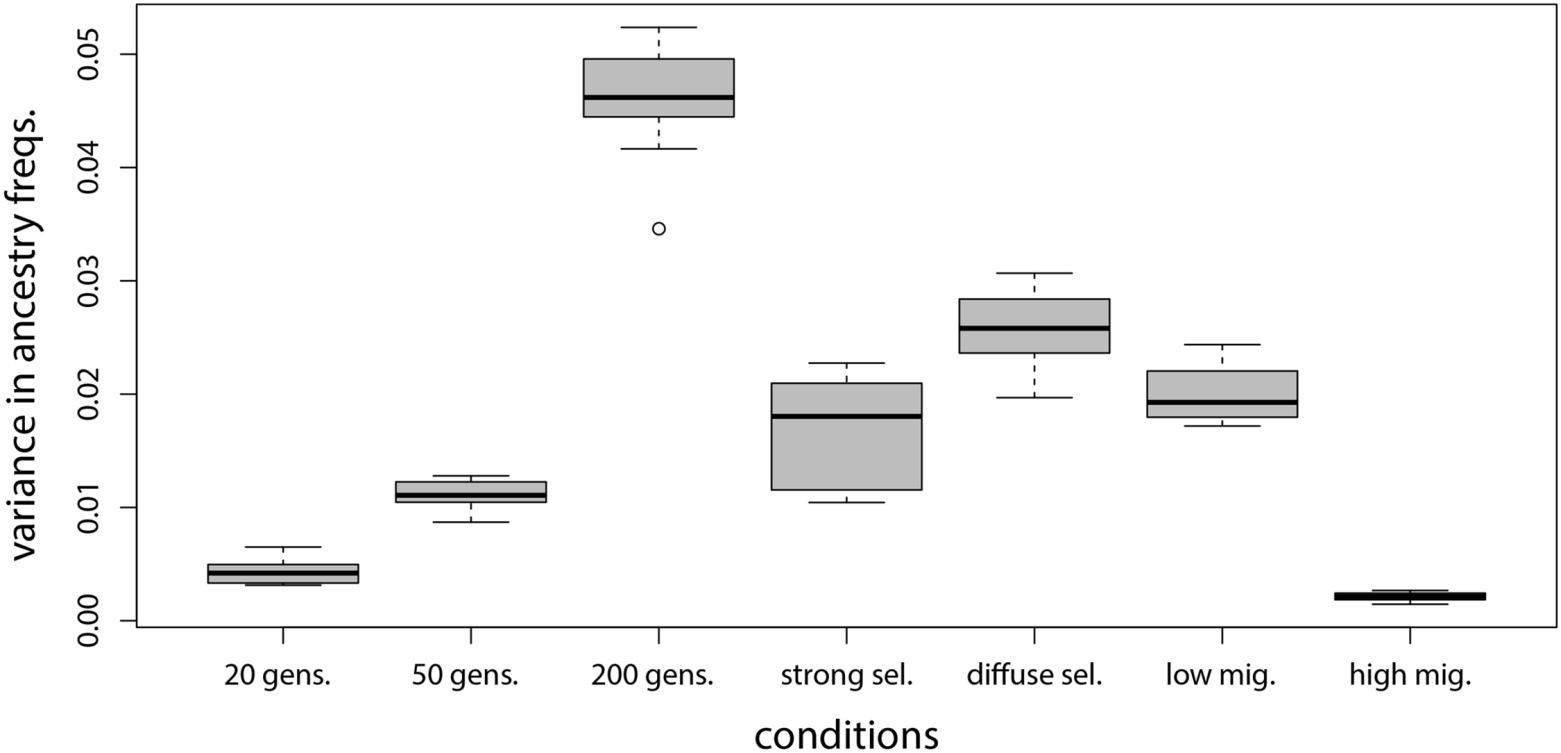
Variation in ancestry frequencies. Boxplots summarize the variance in local ancestry frequencies calculated across 20,002 genetic loci and 10 replicate simulations. This includes 20, 50 and 200 generation simulations without selection, 50 generation simulations with strong or diffuse selection, and 200 generations with low (m = 0.005) or high (m = 0.05) migration.

Variation in local ancestry increased with time and selection, as expected (because dynamics depend on $\frac{t}{N}$, variation in ancestry would also scale with population size, but this was kept constant; [21, 44]). Specifically, ancestry frequencies varied most across the genome in the 200 generation simulations, followed by the 50 generation simulations with diffuse and strong selection; gene flow reduced variation in ancestry frequencies (Fig 2). However, the simulated populations were generally segregating for local ancestry across the genome. In particular the mean proportion of genetic loci where ancestry from one source population reached fixation varied from 0.0% (20 and 50 generation simulations without selection or with diffuse selection) to 0.3% (s.d. = 0.2%; 200 generation simulations) or 2.3% (s.d. = 1.5%; 50 generation simulations with strong selection).

**Fig 3:**
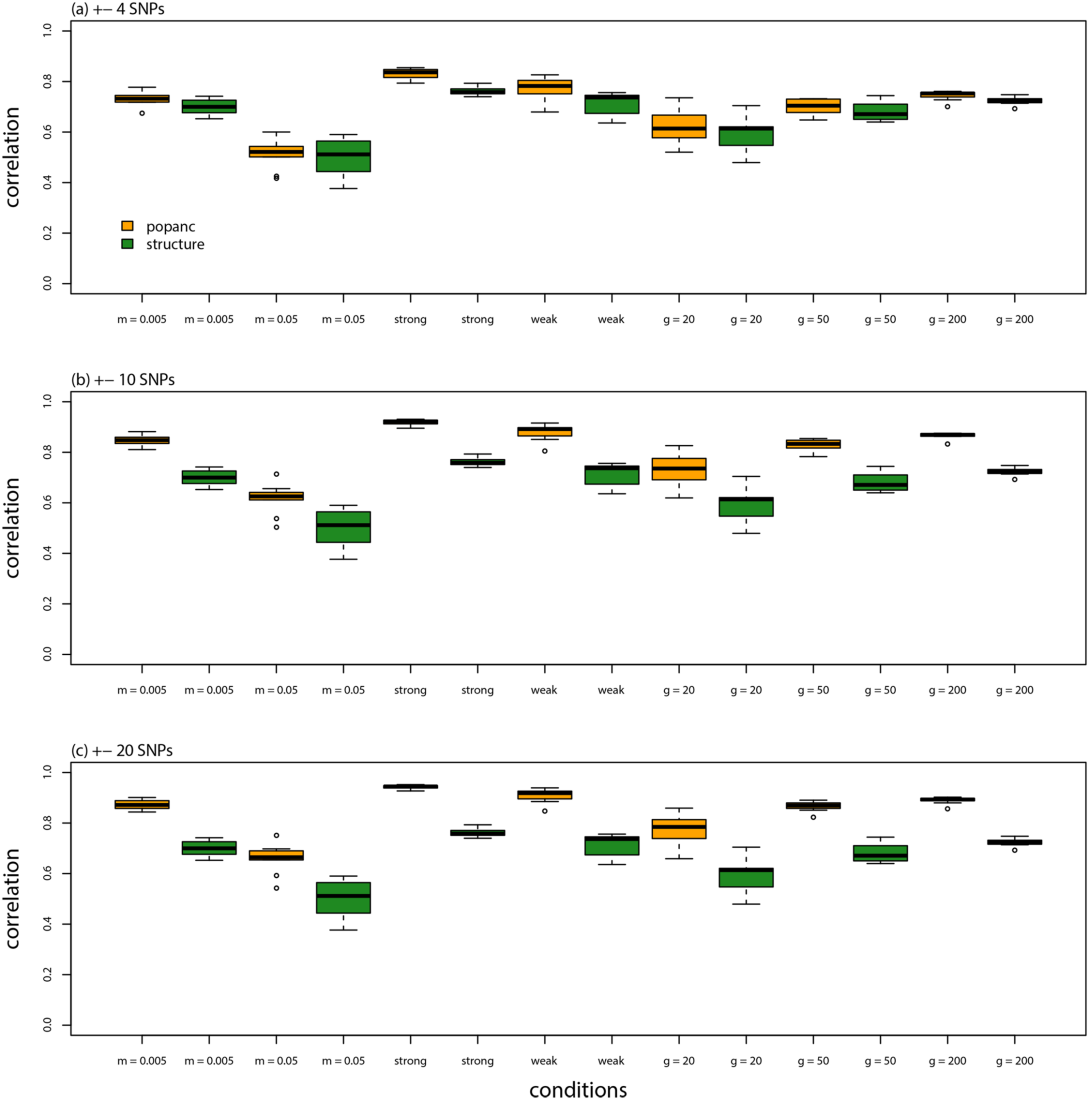
Model evaluation. Boxplots show the distribution of correlations (Pearson correlation coefficient) between true and inferred ancestry frequencies from each of 10 replicate simulations analyzed with different methods, window sizes (for popanc), generations since admixture, selection regimes, and migration rates.

**Fig 4:**
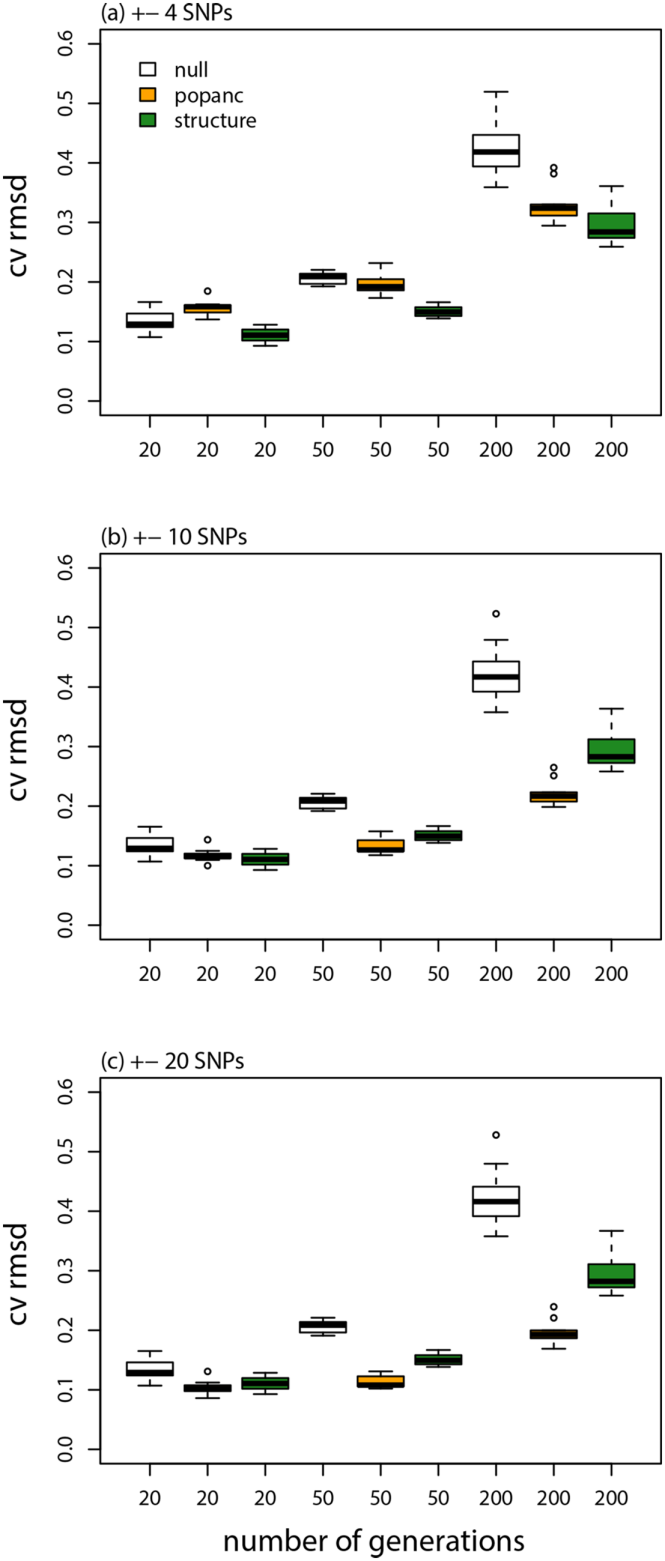
Model evaluation. Boxplots show the average CVRMSD for local ancestry frequencies from each of 10 replicate simulations analyzed with different methods, generations since admixture, and window sizes (for popanc). The CVRMSD for a null model that equates the ancestry frequencies at each locus with the genome-average is also shown.

Accuracy was affected by window size and time since admixture, such that ancestry frequencies were estimated more accurately with larger windows. And there was a greater correlation between true and estimated ancestry frequencies with more time since admixture, but the CVRMSD was lower when admixture occurred more recently (e.g., *t* = 20, ±4 SNPs, *r* = 0.62, CVRMSD = 0.16 vs. *t* = 20, ±20 SNPs, *r* = 0.77, CVRMSD = 0.10 vs. *t* = 200, ±20 SNPs, *r* = 0.89, CVRMSD = 0.20; Figs 3, 4, & 5; Table 1). The HMM in structure performed similarly to or slightly better than the proposed method with a window size of ±4 SNPs, but the proposed CCBPM out-performed this method when windows of ±10 or ±20 SNPs were used, particularly when admixture occurred 200 generations ago (Fig 4; Table 1). Both the proposed CCBPM and the structure HMM were considerably more accurate than the null model of no ancestry frequency variation (Fig 4). Time since admixture and window size also affected coverage of the true parameter value by the 95% ETPIs, with better coverage for more recent admixture and smaller windows (e.g., *t* = 20, ±4 SNPs, coverage = 64% vs. *t* = 200, ±4 SNPs, coverage = 52% vs. *t* = 200, ±20 SNPs, coverage = 41%).

**Fig 5:**
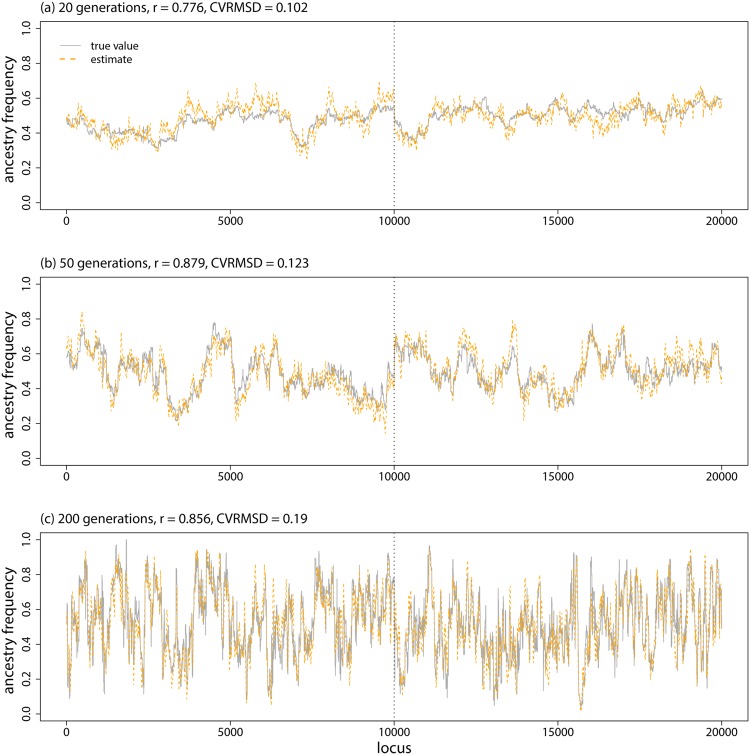
Genetic ancestry estimates from simulated data. Plots show the true and inferred (posterior mean from popanc) ancestry frequencies from one representative data set from the 20, 50, and 200 generation simulations. Results with a window size of ±20 SNPs are shown. The dashed vertical lines delineate the two distinct chromosomes.

**Table 1:** Performance of methods on simulated data. Results shown for local ancestry frequencies estimates in 20, 50, or 200 generation (gens.) simulations with or without selection or ongoing gene flow and analyzed with the proposed method (popanc) or the HMM in structure: Correlation between true and estimated parameter (correlation), normalized RMSD (CVRMSD), and proportion of cases where the true value was included in the 95% ETPIs (95$ ETPI cov.).

gens.	selection	migration	method	correlation (*r*)	CVRSMD	95% ETPI cov.
20	none	none	popanc ±4 SNPs	0.62	0.16	0.64
20	none	none	popanc ±10 SNPs	0.73	0.12	0.56
20	none	none	popanc ±20 SNPs	0.77	0.10	0.60
20	none	none	structure HMM	0.60	0.11	NA
50	none	none	popanc ±4 SNPs	0.70	0.20	0.61
50	none	none	popanc ±10 SNPs	0.83	0.13	0.51
50	none	none	popanc ±20 SNPs	0.87	0.11	0.56
50	none	none	structure HMM	0.68	0.15	NA
200	none	none	popanc ±4 SNPs	0.74	0.33	0.52
200	none	none	popanc ±10 SNPs	0.87	0.22	0.46
200	none	none	popanc ±20 SNPs	0.89	0.20	0.41
200	none	none	structure HMM	0.72	0.29	NA
50	diffuse	none	popanc ±4 SNPs	0.77	0.17	0.61
50	diffuse	none	popanc ±10 SNPs	0.88	0.11	0.51
50	diffuse	none	popanc ±20 SNPs	0.91	0.10	0.56
50	diffuse	none	structure HMM	0.72	0.16	NA
50	strong	none	popanc ±4 SNPs	0.83	0.21	0.60
50	strong	none	popanc ±10 SNPs	0.92	0.14	0.48
50	strong	none	popanc ±20 SNPs	0.94	0.12	0.53
50	strong	none	structure HMM	0.76	0.23	NA
200	none	0.005	popanc ±4 SNPs	0.73	0.24	0.58
200	none	0.005	popanc ±10 SNPs	0.85	0.16	0.47
200	none	0.005	popanc ±20 SNPs	0.87	0.15	0.48
200	none	0.005	structure HMM	0.70	0.21	NA
200	none	0.05	popanc ±4 SNPs	0.51	0.14	0.67
200	none	0.05	popanc ±10 SNPs	0.62	0.11	0.61
200	none	0.05	popanc ±20 SNPs	0.66	0.10	0.64
200	none	0.05	structure HMM	0.50	0.09	NA

**Fig 6:**
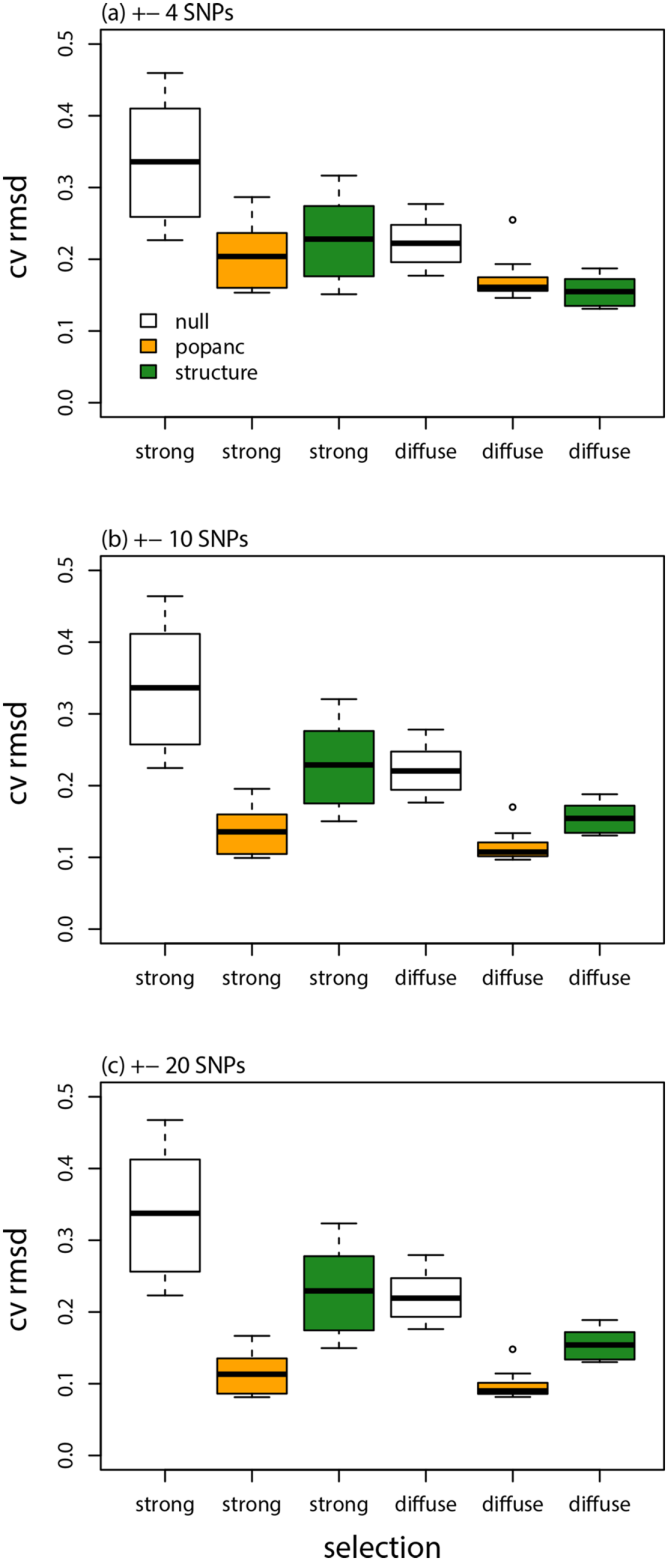
Model evaluation with selection. Boxplots show the average CVRMSD for local ancestry frequencies for each of 10 replicate simulations analyzed with different methods, selection regimes, and window sizes (for popanc). The CVRMSD for a null model that equates the ancestry frequencies at each locus with the genome-average is also shown.

**Fig 7:**
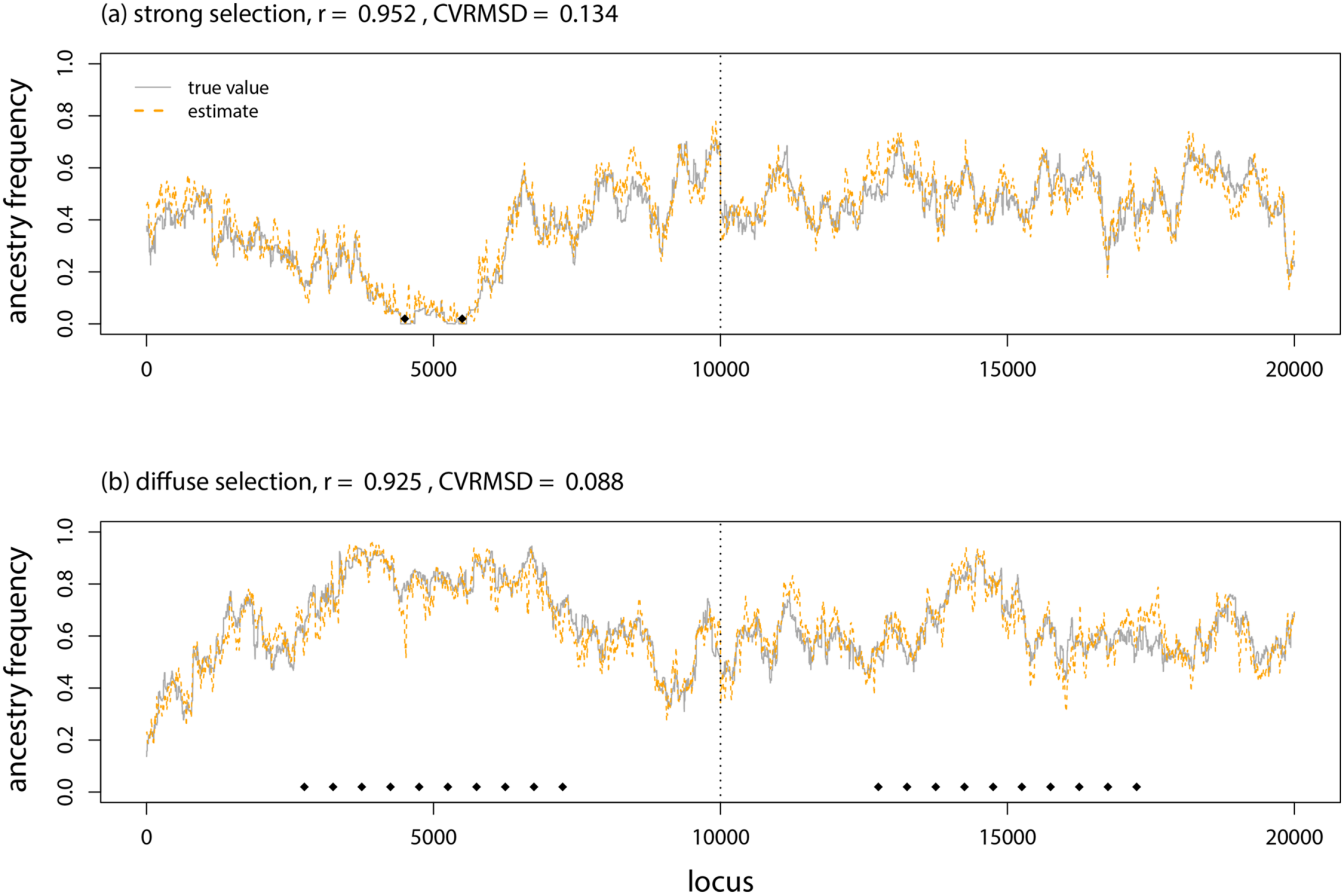
Genetic ancestry estimates from simulated data with selection. Plots show the true and inferred (posterior mean from popanc) ancestry frequencies from one representative data set from the strong and diffuse selection simulations. Results with a window size of ±20 SNPs are shown. The dashed vertical lines delineate the two distinct chromosomes. Black diamonds indicate the genome positions that affected fitness (i.e., the direct targets of selection against inter-population ancestry).

Results with selection were generally similar, but the improved performance of the proposed CCBPM relative to the linkage HMM in structure was more evident (Figs 6 & 7; Table 1). Once again, more accurate estimates were obtained with larger SNP windows. Parameter estimates were more strongly correlated with their true values when selection was strong (e.g., *r*: diffuse selection, ±4 SNPs = 0.77, ±20 SNPs = 0.91; strong selection, ±4 SNPs = 0.83, ±20 SNPs = 0.94; Fig 3; Table 1), but the CVRMSD was lower when selection was diffuse (e.g., CVRMSD: diffuse selection, ±4 SNPs = 0.17, ±20 SNPs = 0.097; strong selection, ±4 SNPs = 0.21, ±20 SNPs = 0.12; Fig 6; Table 1). Nearly identical results were obtained when the SNPs nearest to each selected locus were removed from the analysis (performance metrics were indistinguishable from those in Table 1), thus the improved performance of the CCBPM was not driven by these loci but rather by the overall effect of selection on variation in ancestry frequencies across the genome.

**Fig 8:**
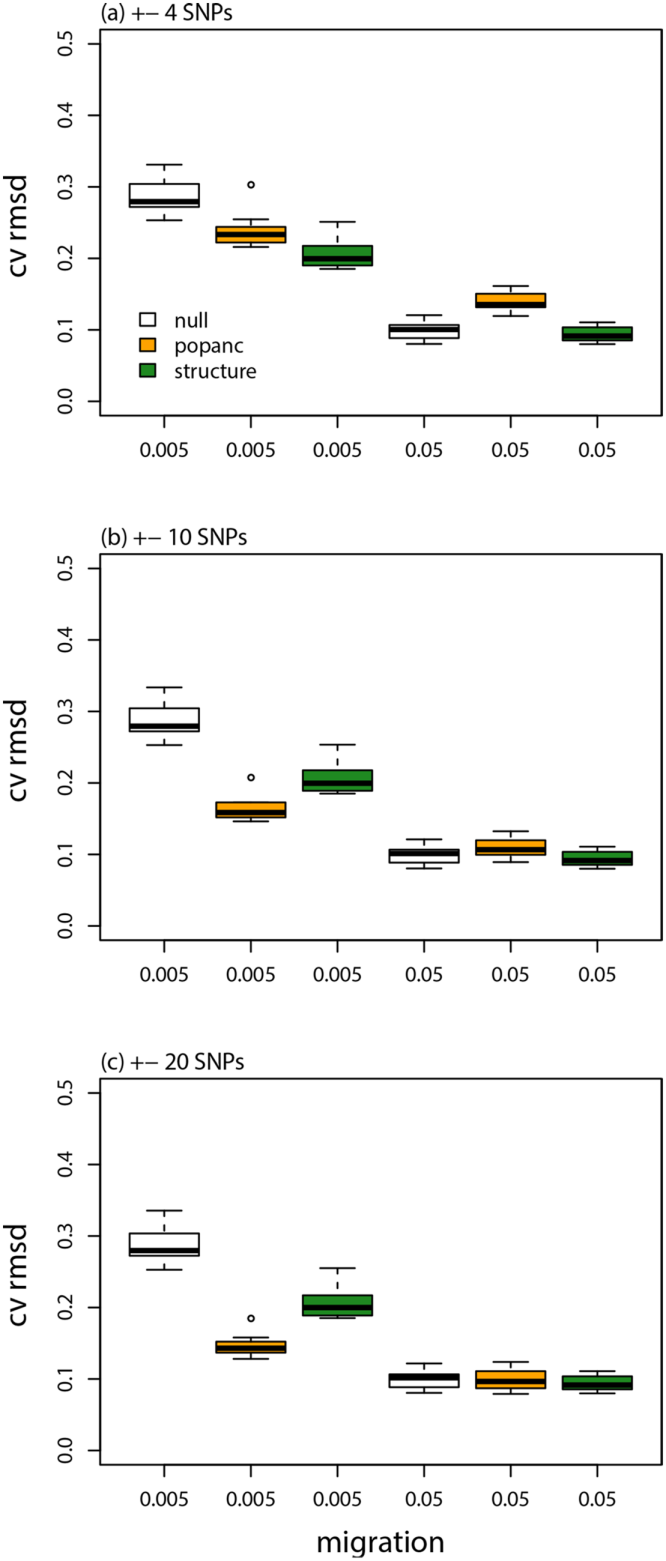
Model evaluation with on-going gene flow. Boxplots show the average CVRMSD for local ancestry frequencies for each of 10 replicate simulations analyzed with different methods, migration rates, and window sizes (for popanc). The CVRMSD for a null model that equates the ancestry frequencies at each locus with the genome-average is also shown.

A low rate of ongoing gene flow after admixture (i.e., *m* = 0.005) did not noticeably degrade the performance of the CCBPM in general or relative to the linkage HMM in structure (Figs 3, 8, & 9; Table 1). However, parameter estimates were less strongly correlated with their true values when ongoing gene flow occurred at a higher rate (*r*: ±4 SNPs = 0.51, ±10 SNPs = 0.62, ±20 SNPs = 0.66). But, even under these conditions, ancestry estimates from the CCBPM were more strongly correlated with their true values than the estimates from the HMM, particularly when a window size of ±10 or ±20 was used (Fig 3; Table 1).

**Fig 9:**
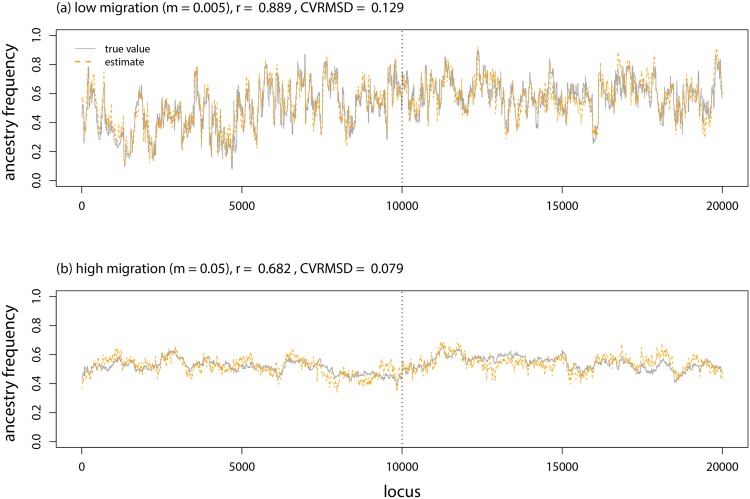
Genetic ancestry estimates from simulated data with on-going gene flow. Plots show the true and inferred (posterior mean from popanc) ancestry frequencies from one representative data set from simulations with low or high gene flow. Results with a window size of ±20 SNPs are shown. The dashed vertical lines delineate the two distinct chromosomes.

### Admixture in humans

**Fig 10:**
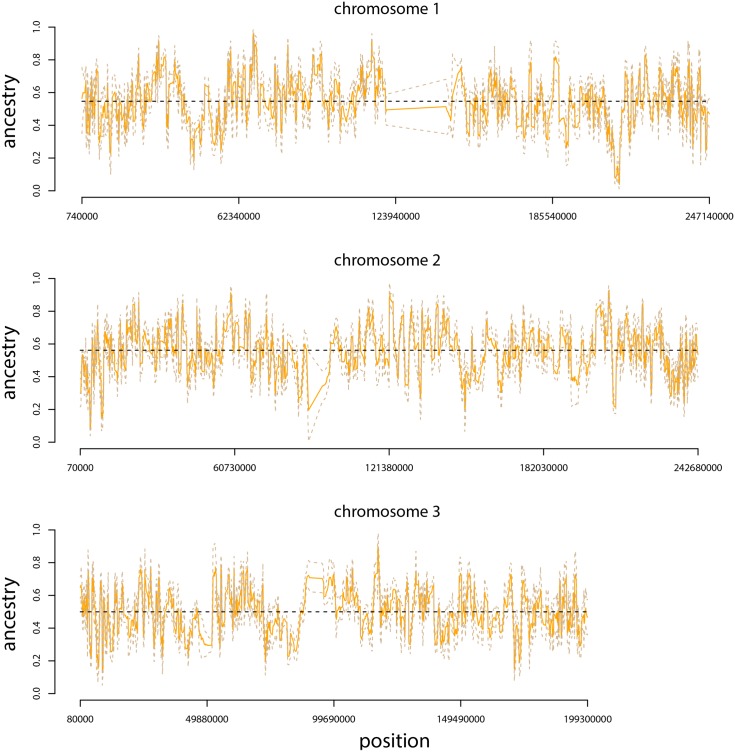
Genetic ancestry in Uyghur. Plots show ancestry frequency estimates from three human chromosomes. The posterior median (solid, orange line) and 95% ETPIs (dashed, tan line) for Han ancestry are given. The average ancestry frequency for each chromosome is shown by the dashed, black line.

The genome-average ancestry frequencies in the Uyghur population were 52.9% Han and 47.1% French, which is consistent with [7]. Average ancestry frequencies varied modestly among chromosomes, ranging from 47.0% Han on chromosome 18 to 58.6% Han on chromosome 20. Even more variation in ancestry frequencies was observed within chromosomes (average ancestry frequency variance within chromosomes = 0.016, s.d. = 0.003; Fig 10). Indeed, for 55.1% of loci, the chromosome-average ancestry frequency was outside of the local ancestry frequency. Although no regions of fixed ancestry were detected, ancestry frequencies as high as 96.1% Han or 95.6% French were observed.

Genetic loci with the greatest excess or deficit of Han or French ancestry, that is, the 118 SNPs below the 0.05^th^ (9.0% Han) or above the 99.95^th^ (92.7% Han) empirical quantile for ancestry frequencies, were analyzed in more detail. These 118 SNPs formed three contiguous genetic regions with excess Han ancestry (two on chromosome 2 and one on chromosome 10) and four contiguous regions with excess French ancestry (one on chromosome 2 and three on chromosome 11; mean size of region = 408.9 kbp; Table 2). Most of these genetic regions contain one or more known genes. For example, a region of excess French ancestry on chromosome 11 includes *bridging integrator 1* (*BIN1*), and variation in this gene has repeatedly been associated with Alzheimer’s disease [70–72]. We also found evidence consistent with the hypothesis of selection on this gene in the French: *F*_*ST*_ between French and Han was elevated for the two SNPs in BIN1 relative to the rest of chromosome 2 (*F*_*ST*_ for BIN1 = 0.23, mean for chromosome 2 = 0.073, randomization test, *p* = 0.0617), and genetic diversity was significantly reduced in the French population (heterozygosity for BIN1 = 0.00, mean for chromosome 2 = 0.37, randomization test, *p* < 0.0001).

**Table 2:** Top excess ancestry regions in the Uyghur. Chromosome and position (start and end) for seven regions of excess Han or French ancestry in the Uyghur. Genes in these regions were identified using the UCSC Genome Browser on the Human Feb. 2009 (GRCh37/hg19) Assembly.

chrom.	start (kbp)	end (kbp)	no. SNPs	excess	genes
2	155508	157032	43	Han	KCNJ3
2	182130	182175	3	Han	AK125001
10	72176	72303	13	Han	EIF4EBP2, NODAL, PALD1
2	127590	127875	17	French	BIN1
11	26727	27268	24	French	BBOX1, FIBIN, SLC5A12
11	28373	28657	16	French	none
11	89859	89915	2	French	NAALAD2

### Mouse hybrid zone

**Fig 11:**
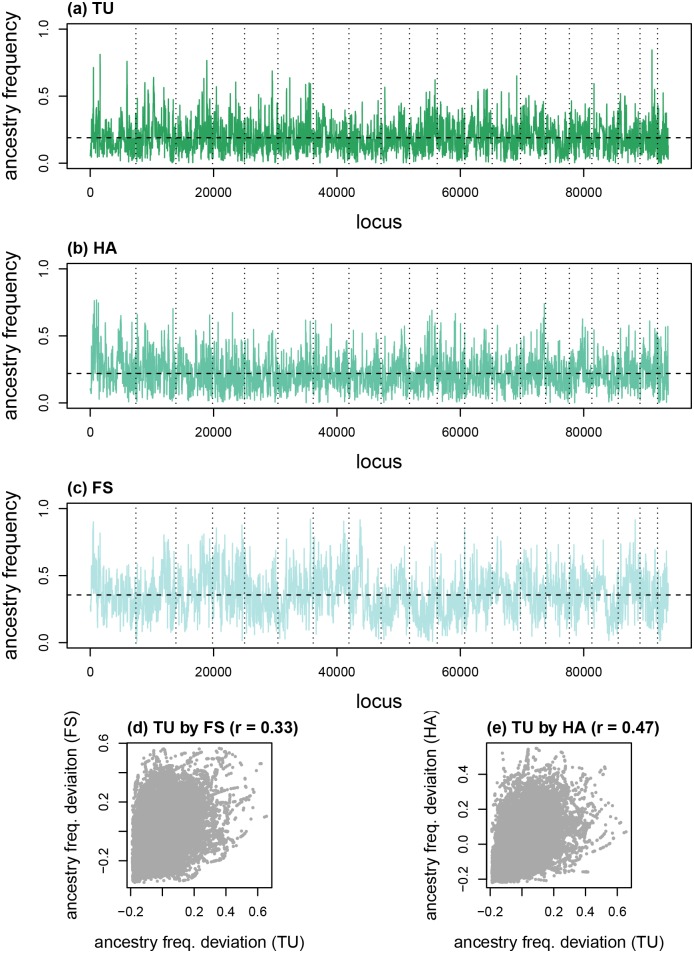
Genetic ancestry in a mouse hybrid zone. Plots show ancestry frequency estimates from three admixed populations. Solid lines in (a-c) give ancestry frequencies along chromosomes for TU, HA, and FS populations. Vertical dotted lines delineate chromosomes and the average ancestry frequency for each chromosome is shown by the dashed, black line. Scatterplots in (e) and (f) depict correlations in ancestry frequencies (relative to the mean for each population) for pairs of populations.

Genome-average ancestry frequencies varied among admixed mouse populations from 19.0% (s.d. = 9.5%) and 21.9% (s.d. = 10.4%) *M. domesticus* in TU and HA to 35.6% (s.d. = 13.7%) *M. domesticus* in FS (Fig 11). This differed somewhat from, but were correlated with previous estimates of hybrid index which were based on different reference samples and different SNP markers [67]. As with the Uyghur, average ancestry frequencies differed substantially among chromosomes in each admixed population (ranges: TU = 13.8% *M. domesticus* to 22.5%, HA 18.6% to 26.9%, FS = 25.1% to 45.4%). In each population, some genetic loci were fixed or nearly fixed for *M. musculus* ancestry (number of genetic loci with *M. domesticus* ancestry frequencies < 1%: TU = 202, HA = 96, FS = 2), while maximum frequencies of *M. domesticus* ancestry were high, but always less than one (TU = 84.5%, HA = 76.8%, FS = 92.1%).

Ancestry frequencies were correlated between pairs of admixed populations (TU × HA: *r* = 0.47, *p* < 0.001; TU × FS: *r* = 0.33, *p* < 0.001; HA × FS: *r* = 0.33, *p* < 0.001; Fig 11). Moreover, several of the same genetic loci had high *M. domesticus* ancestry in more than one admixed population. For example, considering the 0.5% of SNPs with the highest *M. domesticus* frequency in each admixed population (469 SNPs), 173 high *M. domesticus* ancestry SNPs were shared between two populations and six were shared among all three admixed populations. This is significantly more overlap than would be expected under a null hypothesis of independence among populations (null for two pops.: expected = 7.4, ×-fold enrichment [observed/expected] = 23.3×, *p* < 0.001; null for all three pops.” expected < 0.01, ×-fold enrichment = 1500×, *p* < 0.001). However, because of gene flow, strict independence would not be expected anyway. A similar analysis was not conducted for SNPs with excess *M. musculus* ancestry, because TU and HA had high frequencies of *M. musculus* ancestry overall and were fixed or nearly fixed for *M. musculus* ancestry at dozens of SNPs (as described in the previous paragraph). Consistent results were found when considering the 0.05% SNPs with the greatest excess of *M. domesticus* ancestry (47 SNPs above the 99.95^th^ empirical quantile) in each population (Table 3). While none of these were shared among populations, neighboring regions of excess *M. domesticus* were detected in the three populations (chromosome 1, 21, 910–22, 026 kb in TU, 20, 0825–21, 726 kb in FS, and 24,848–25, 420 kb in HA). Moreover, regions with the greatest excess *M. domesticus* ancestry in any one population had elevated *M. domesticus* ancestry frequencies in the others (randomization test, TU SNPs: mean in other pops. = 0.455, ratio of observed to null expectation = 1.58×, *p* = 0.002; HA SNPs: mean in other pops. = 0.417, ratio of observed to null expectation = 1.54×, *p* = 0.012; FS SNPs: mean in other pops. = 0.392, ratio of observed to null expectation = 1.91×, *p* < 0.001).

**Table 3:** Top excess ancestry regions in house mice. Chromosome and position (start and end) for regions with the greatest excess *M. domesticus* ancestry in each of the three admixed house mouse populations. Only regions comprised of more than one SNP were reported.

population	chrom.	start (kbp)	end (kbp)	no. SNPs
TU	1	21910	22026	8
TU	1	47056	47096	3
TU	1	168139	168693	18
TU	3	139005	139401	15
TU	19	44236	44237	2
HA	1	24848	25420	26
HA	1	35230	35298	6
HA	1	40639	40774	9
HA	2	170032	170038	3
HA	14	120403	120433	4
FU*	1	20825	21726	23
FU	6	141892	142305	10
FU	7	138382	138450	3
FU	8	46928	46988	9
FU	18	73871	73891	2

* this range represents a composite of a few nearly contiguous regions.

## Discussion

### Performance and utility

I have described a new method to infer local ancestry frequencies from un-phased genotypic data using a continuous correlated beta process model (CCBPM). The proposed method produced accurate estimates of ancestry frequencies, outperforming a traditional HMM (and a null model of no variation in ancestry frequencies) under most conditions examined (e.g., Figs 3, 4, 6, & 8, Table 1). As expected, the improved performance of this new approach relative to the HMM in structure was more pronounced when ancestry frequencies varied more across the genome, either because of selection, more ancient admixture, or little to no gene flow post admixture. The poorer performance of the homogeneous HMM under these conditions likely reflects the fact that the HMM makes the *a priori* assumption that ancestry frequencies do not vary. This should bias local ancestry inference towards the genome-average unless the genetic data are perfectly informative of ancestry. Moreover, the difference in performance between these methods was not trivial; for example, the CVRMSD for ancestry frequencies in the 200 generation simulations with ±20 SNP windows for the CCBPM was only $∼\frac{2}{3}$ that of the structure HMM. Another key distinction between the approaches is that CCBPM generates measures of uncertainty in the ancestry frequencies (as captured in the approximate posterior distribution), which account for uncertainty in local ancestry within individuals, whereas *post hoc* estimates of ancestry frequencies from deconvolutions of local ancestry within individuals do not. However, this benefit is lessened by the fact that the 95% ETPIs inferred from the CCBPM appear to routinely underestimate uncertainty in local ancestry (the cause and a potential solution for this are discussed more below; Table 1). Finally, computational methods used for the proposed method are relatively efficient, and thus run times should not be prohibitive for the analysis of large GBS, SNP, or even whole genome sequence data (for the results presented here runs on standard Linux compute nodes took 2-5 hours, and different chromosomes can be analyzed separately).

By applying the proposed method to a case of historical admixture in a human population, I further documented the reliability and utility of the approach. The method generated estimates of genome-average ancestry consistent with previous studies [7], but also showed that ancestry frequencies varied substantially both within and among chromosomes (Fig 10; [60] also reported chromosome-average ancestries which were similar to those obtained with popanc, *r* = 0.895, *p* < 0.0001). Ancestry frequency variation in the Uyghur likely reflects the effects of drift and selection, but parsing these effects remains difficult, as drift can have a substantial effect on ancestry frequencies given sufficient time (e.g., Fig 1). Here, comparisons with other admixed human populations could be useful, as selection would be more likely to generate consistent excess ancestry in the same regions of the genome (e.g., [73–75]). Analyzing such variation in ancestry can also be important for uncovering the basis for variation in traits and the prevalence of diseases among different human populations (e.g., [26, 27, 76–79]). Thus, ancestry frequency inference can be viewed as complementary to admixture mapping approaches used in recently admixed populations (e.g., African Americans or Hispanic or Latino populations), which utilize variation in local ancestry blocks and disease risk among individuals within a population (e.g., [26, 80, 81]). Along these lines, here I found a 285 kb region on chromosome 11 in the Uyghur population where greater than 90% of gene copies harbored French ancestry, whereas the genome-average frequency of French ancestry was only 47.1%. This region also contained a gene that has been repeatedly associated with Alzheimer’s risk [70, 71], and showed patterns of genetic differentiation and variation in the source populations consistent with a history of selection in the French. Thus, it is possible that risk for this or a related disease was affected by this gene and varied between the ancestral source populations of the Uyghur, though further work would be required to test this hypothesis.

The proposed CCBPM was also applied to genetic data from three admixed populations that were part of the Bavarian transect through the central European *M. domesticus* × *M. musculus* hybrid zone. As with the human data, notable variation in ancestry frequencies was detected within and among chromosomes. Genetic regions with high or very high *M. domesticus* ancestry frequencies in one population tended to have higher *M. domesticus* ancestry frequencies in the other populations as well. Often such patterns of consistency or parallelism across replicate populations are interpreted as evidence of selection [14, 73]. While this could be correct here, gene flow among admixed populations could also explain this pattern. Indeed, comparative analyses of distant transects through this hybrid zone have shown that patterns of introgression vary considerably in different parts of this hybrid zone [68]. Of course, gene flow and selection are not mutually exclusive hypotheses. And additional data support the hypothesis that at least one of the excess *M. domesticus* ancestry regions (chromosome 1, 168, 139–168, 693 kb in TU) was likely affected by selection as it has been associated with a trait (aberrant RNA expression patterns in the testis) that likely contributes to reduced fertility in hybrids [69], and coincides with a marker with a putative epistatic effect on fitness in this hybrid zone [82].

Results from these empirical studies raise an important question: to what extent can genomic variation in ancestry frequencies be interpreted as evidence for past selection? Clearly, selection can drive extreme ancestry frequencies (Fig 7; [39, 44]). In particular, if selection in an admixed population favors a generally beneficial allele that had a higher frequency in one source population, the local ancestry frequency of the chromosomal segment containing that allele should increase. Underdominance and epistasis will have a similar effect, but the favored allele and ancestry type should exhibit positive frequency dependence, and thus, the outcome will depend on the initial conditions (e.g., with underdominance the marginal fitness of the more common ancestry type will be higher because it will occur proportionally less often in heterozygotes; [44]). However, genetic drift, particularly in small or old admixed populations, can also cause substantial variation in ancestry frequencies (Fig 5). The effects of drift and selection can be hard to disentangle, particularly when selection is weak. This could be further confounded by variation in recombination rates, which would cause some genetic regions to be more or less influenced by the indirect effects of selection [83–85]. This will be particularly pronounced in admixed populations, because of admixture linkage disequilibrium. Thus, while it would be possible to develop an explicit test for selection-based patterns of ancestry frequency variation (e.g., [39, 40]), I avoid that here. Rather, genetic regions with extreme ancestry frequencies (relative to the overall variance across the genome) should be viewed as potential regions of interest that are likely enriched for targets of selection, but one cannot assume that all or most of these have in fact experienced substantial selection. Contrasts among independent admixed populations (e.g., [68, 86, 87]) or between admixed and allopatric source populations (e.g., [18, 88]) could be used to further test the hypothesis of selection, but this often assumes selection acts similarly across populations and environments.

### Methodological considerations

As demonstrated here, window size affects the accuracy of ancestry inference. In particular, because of drift in admixed populations and shared variation between source populations, information must be extracted from a series of SNPs to obtain accurate estimates of local ancestry and ancestry frequencies (hence the popularity of window-based methods for local ancestry inference, e.g., [34, 55, 89]). Thus, for the simulated data sets analyzed here, ancestry frequencies were better estimated with the larger ±10 and ±20 SNP windows than the ±4 SNP windows (Figs 4 & 6, Table 1). However, very large windows will also lead to errors, because windows will frequently span ancestry breakpoints, and thus will include a mixture of SNPs with different ancestry. Note that window size and the scale parameter from the kernel function are related but distinct. Window size reflects ancestry blocks within individuals, whereas the scale parameter captures autocorrelation in ancestry frequencies at the population-level and is inferred from the data.

A few different approaches could be used to select an appropriate window size. First, if the time since admixture is known, the expected density of ancestry breakpoints and thus size of ancestry blocks can be calculated. For example, if one assumes that recombination operates as a random Poisson process and that half of all recombination events cause ancestry breakpoints (ancestry breakpoints are only generated by recombination between chromosomes with different local ancestry [11]), then the density of ancestry breakpoints for a pair of homologous chromosomes (i.e., in a diploid individual) should be $\frac{L^{chrom}}{t}$, where *L*^*chrom*^ is the map size of the chromosome in Morgans (M) and *t* is the time since admixture. Assuming one has genotypes from *L*^*SNP*^ SNPs on each chromosome, and that each chromosome is 1 M in length, ancestry blocks should thus contain an average of $\frac{L^{SNP}}{t}$ SNPs. Therefore, window sizes smaller than $\frac{L^{SNP}}{t}$ SNPs should be used. When admixture is very old or the contributions of the source populations differ substantially, this equation will underestimate ancestry block size and thus the approach is conservative (this occurs because recombination is less likely to generate breakpoints when ancestry frequencies are farther from 0.5). Applying this equation to the 20, 50 and 200 generation simulations analyzed here, one would expect average ancestry block sizes of 500 (*t* = 20), 200 (*t* = 50), and 50 (*t* = 200) SNPs. As expected, these numbers slightly underestimate the true block sizes, which were 540.3 (*t* = 20), 213.0 (*t* = 50), and 59.4 (*t* = 200) SNPs. Alternatively, if the time since admixture is not known, one can obtain a reasonable estimate of the size of ancestry blocks by first inferring local ancestry using a HMM, such as the linkage model in structure [32], or one of the other available HMMs (e.g., [33, 36]). Moreover, data sets can be analyzed using a series of window sizes to evaluate the robustness of the results to this parameter. One should then be able to identify a range of reasonable window sizes that give consistent estimates of local ancestry frequencies.

The proposed method uses DA to calculate the likelihood of local ancestry given the genetic data, that is, Pr(*s*_*xj*_|*z*_*xj*_) (Eq 6). However, the proposed CCBPM could be combined with ancestry likelihoods obtained from other discriminant methods, such as the random forest algorithm implemented in RFMix [37]. With that said, DA is rapid and can be used for integer or non-integer valued genetic data. The latter is a particularly nice feature, as this means that posterior mean genotypes, such as those obtained from imputation or low to moderate coverage GBS data [6, 90, 91], can readily be handled by the proposed method with DA. Thus, this is an important feature to ensure that the proposed method can be applied to non-model systems.

A final issue of note is that the approximate 95% ETPIs for local ancestry were consistently too narrow, and thus often failed to contain true parameter value; this was true despite low overall errors and high correlations between parameter values and their estimates (Table 1). This is a predictable outcome of the generalized Bayesian update for the CCBPM specified in Eq (1). By combining information across genetic loci, the CCBPM generates more accurate estimates of ancestry frequencies that account for autocorrelations along chromosomes, but this also unduly narrows the posterior distribution for these parameters. This problem could be circumvented by obtaining point estimates from the CCBPM posterior distribution (i.e., by using Eq (1)), but also sampling from beta(*α* = *z*_*x*_ + *α*_0_, *β* = 2*n*_*x*_ − *z*_*x*_ + *β*_0_) to obtain 95% ETPIs for that parameter. This option is available in the popanc software, and greatly increased the proportion of the time the 95% ETPIs contained the true parameter value for a strong selection data set that I re-analyzed (95% ETIP coverage: CCBPM = 0.48, simple beta-binomial model = 0.78).

### Conclusions

Existing ancestry deconvolution methods are best suited for relatively recent or very ancient admixture. In the former case, ancestry frequencies should not vary much across the genome and instead variation in genome-average ancestry or local ancestry-blocks are of interest, whereas in the latter case one can assume that admixed population or species are fixed for ancestry blocks. However, admixed populations between these two extremes exist [6], and should exhibit substantial variation in ancestry frequencies (Fig 1). The method proposed and evaluated in this paper can be used to estimate ancestry frequencies under such conditions. Ancestry frequencies can then be examined to describe the genomic composition of admixed populations and to evaluate progress towards genome stabilization (i.e., the loss of segregating variation for local ancestry). Moreover, comparisons of admixture frequencies across multiple admixed populations could provide critical information on whether hybridization has repeatable, predictable outcomes, and thus on the relative roles of deterministic and stochastic processes in shaping genome composition [92]. Additionally, admixture mapping methods could condition on local ancestry frequencies and thereby gain power to map important trait variation in admixed populations. And finally, ancestry frequencies could help identify regions of the genome that have been targets of selection in hybrids, which is particularly relevant for understanding the genetic basis of reproductive isolation between incipient species [15, 39, 93]. Thus, by filling what has been an analytical gap, the proposed CCBPM should be a useful tool for a variety of biologists.
